# The effect of interruption to propofol sedation on auditory event-related potentials and electroencephalogram in intensive care patients

**DOI:** 10.1186/cc2984

**Published:** 2004-10-22

**Authors:** Heidi Yppärilä, Silvia Nunes, Ilkka Korhonen, Juhani Partanen, Esko Ruokonen

**Affiliations:** 1 Department of Clinical Neurophysiology, Kuopio University Hospital, and Department of Applied Physics, University of Kuopio, Kuopio, Finland; 2 Department of Anesthesiology and Intensive Care, Division of Intensive Care, Kuopio University Hospital, Kuopio, Finland; 3 Professor, VTT Information Technology, Tampere, Finland; 4 Professor, Department of Clinical Neurophysiology, Kuopio University Hospital, Kuopio, Finland; 5 Department of Anesthesiology and Intensive Care, Division of Intensive Care, Kuopio University Hospital, Kuopio, Finland

**Keywords:** electroencephalogram, event-related potentials, intensive care, propofol, sedation

## Abstract

**Introduction:**

In this observational pilot study we evaluated the electroencephalogram (EEG) and auditory event-related potentials (ERPs) before and after discontinuation of propofol sedation in neurologically intact intensive care patients.

**Methods:**

Nineteen intensive care unit patients received a propofol infusion in accordance with a sedation protocol. The EEG signal and the ERPs were measured at the frontal region (Fz) and central region (Cz), both during propofol sedation and after cessation of infusion when the sedative effects had subsided. The EEG signal was subjected to power spectral estimation, and the total root mean squared power and spectral edge frequency 95% were computed. For ERPs, we used an oddball paradigm to obtain the N100 and the mismatch negativity components.

**Results:**

Despite considerable individual variability, the root mean squared power at Cz and Fz (*P *= 0.004 and *P *= 0.005, respectively) and the amplitude of the N100 component in response to the standard stimulus at Fz (*P *= 0.022) increased significantly after interruption to sedation. The amplitude of the N100 component (at Cz and Fz) was the only parameter that differed between sedation levels during propofol sedation (deep versus moderate versus light sedation: *P *= 0.016 and *P *= 0.008 for Cz and Fz, respectively). None of the computed parameters correlated with duration of propofol infusion.

**Conclusion:**

Our findings suggest that use of ERPs, especially the N100 potential, may help to differentiate between levels of sedation. Thus, they may represent a useful complement to clinical sedation scales in the monitoring of sedation status over time in a heterogeneous group of neurologically intact intensive care patients.

## Introduction

The majority of mechanically ventilated patients in the intensive care unit (ICU) require sedation to reduce their anxiety and to increase their tolerance of the tracheal tube and mechanical ventilation. The choice of sedative drugs and the way in which they are administered may have an important impact on patient outcome and cost of care [1]. Excessively deep sedation will prolong ventilator dependence and length of stay in the ICU, which can be avoided by careful monitoring and interruption to sedative infusions [2]. Differentiation between adequate comfort and excessive sedation requires the use of clinically relevant sedation scales; however, these are not suitable for application during deep sedation or muscle relaxation. Other methods to assess the level of sedation in the clinical setting are therefore needed.

Growing knowledge of the depressive effects of sedative drugs on the central nervous system has led to increasing interest in a possible correlation between neurophysiological indices and the level of sedation. The most commonly used neurophysiological indices in the assessment of sedation are electroencephalogram (EEG) and auditory evoked potentials (AEPs), which measure different aspects of brain functioning. The evoked potentials show whether the central nervous system responds systematically to an auditory stimulus, and they may thus be considered a direct measure of the responsiveness of the brain. In contrast, the EEG signal, if not associated with a sensory stimulus, will only reflect the ongoing background electrical activity of the brain. In other words, if the patient is not stimulated and the level of sedation is measured using indices derived from the EEG signal, then it can be speculated that those indices may only be used as predictors of whether the patient will actually react to a given stimulus, but they provide no measure of responsiveness. AEPs may therefore provide a more accurate tool with which to assess the level of sedation.

**Figure 1:**
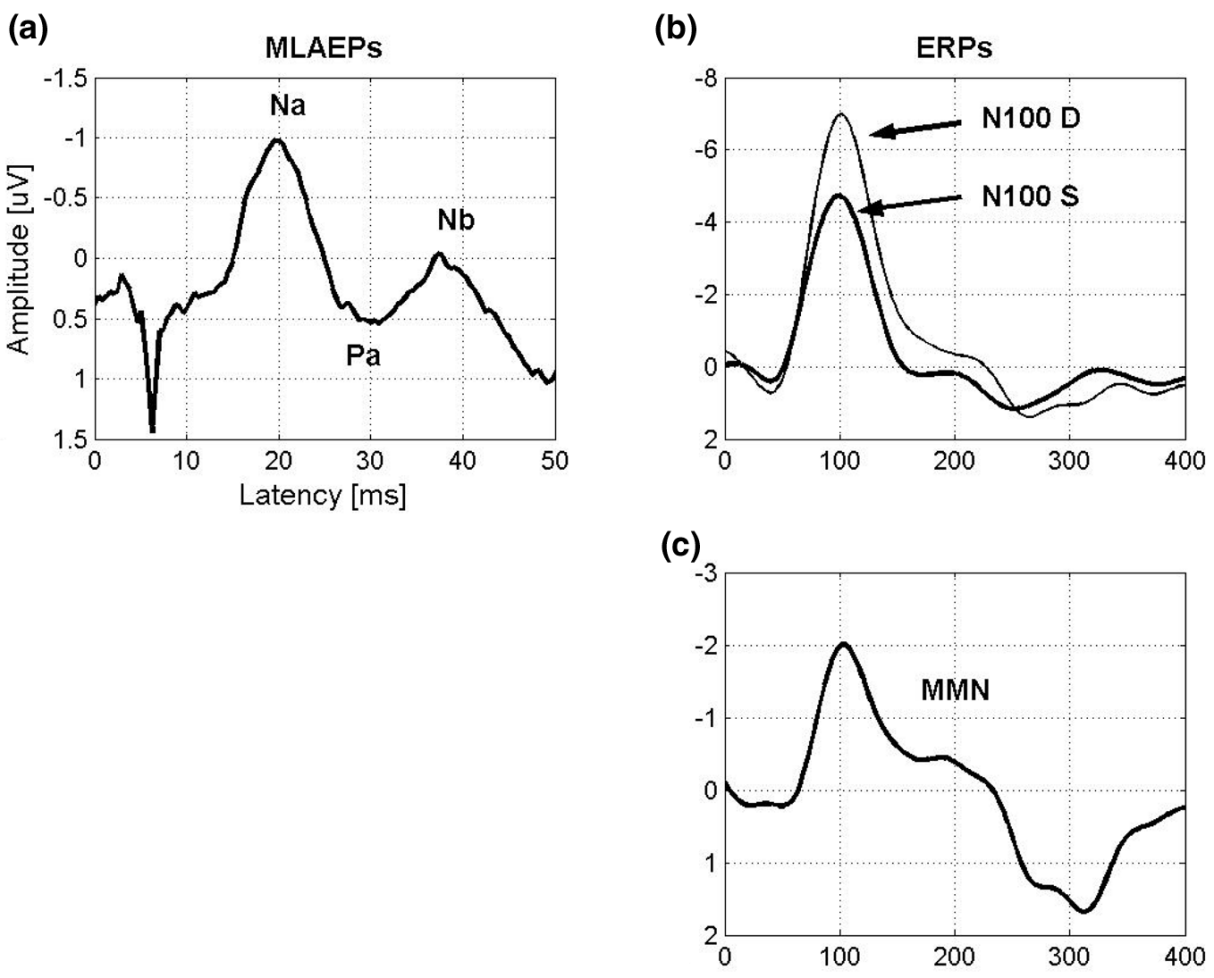
**(a) **The middle-latency auditory evoked potential (MLAEP) components Na, Pa, and Nb appear 10–50 ms after the onset of auditory stimulus. **(b) **N100 is the most prominent event-related potential (ERP) component. The thick line is the N100 for standard stimuli (N100 S) and the thin line is the N100 for deviant stimuli (N100 D). (c) The mismatch negativity (MMN) curve is obtained as a difference curve N100 D–N100 S. The MMN is the negative area under the curve between 100 and 250 ms.

Within the AEPs, the middle-latency AEPs (10–50 ms after the stimulus) are mainly evoked by the physical features of the auditory stimulus. Their presence establishes the integrity of the afferent auditory pathway and confirms that basic auditory signal processing is taking place in the primary auditory cortex (Fig. 1a). The long-latency AEPs, or event-related potentials (ERPs; >50 ms after the stimulus), result from deeper processing of the auditory stimulus and are generated by areas of cortex at and beyond the primary projection area. ERPs may therefore be better indicators of the effect of sedative drugs on the mental state than are middle-latency AEPs.

The most prominent ERP component is N100, which appears about 100 ms after the onset of stimulus and reflects the simultaneous activation of several different brain regions, indicating detection of a change in acoustic surroundings (Fig. 1b) [3]. Another ERP component, namely mismatch negativity (MMN), is elicited by infrequently presented stimuli that differ in some physical dimension from the standard stimuli and reflects the brain's automatic auditory change detection mechanism, which depends on the integrity of auditory sensory memory (Fig. 1c) [4]. Appearance of MMN indicates that several brain regions are activated simultaneously. The fact that MMN reflects widespread brain activation may explain why sedative drug induced changes in the MMN have been shown to be a better marker of mental state than are the respective changes in the middle-latency AEPs [5].

ERPs have exhibited graded changes with increasing doses of sedative drugs in volunteers and surgical patients [6,7], but to date only few data are available concerning the use of ERPs for monitoring sedation level in the ICU. Despite the known superiority of ERP parameters over EEG parameters for monitoring sedation level, in this preliminary pilot study we hypothesized that both ERPs and EEG may be used to assess the level of sedation in a heterogeneous group of neurologically intact intensive care patients.

## Methods

The study protocol was approved by the local ethical committee and written informed consent was obtained from each patient or from the next of kin.

We measured EEG and ERPs in a heterogeneous group (*n *= 19; 13 males and six females; age 65 ± 11 years) of mechanically ventilated patients presenting with a range of surgical and medical conditions requiring intensive care but with no known organic brain dysfunction (Table 1). Patients who were known to have impaired hearing were excluded from the study. Sedation was administered following the modified Brook protocol [1]. Repeated midazolam boluses were initially used to induce and maintain sedation. If the obtained sedation level was still considered inadequate, then propofol infusion was begun and midazolam administration discontinued. The optimal depth of sedation for each patient was determined on clinical grounds, independent of the study, and was assessed using the sedation–agitation scale (SAS; Table 2) [8].

At the time of the first EEG and ERP recordings, patients were receiving propofol sedation (infusion rate 1.91 ± 0.88 mg/kg per hour) and the duration of the infusion had exceeded 8 hours in all patients (31 ± 29 hours). Discontinuation of sedation was then considered necessary so that the patients could be weaned from the ventilator or so that their neurological status could be evaluated. Propofol infusion was interrupted, and the measurements were repeated once the sedation had subsided and the patients were able to follow commands (i.e. to open their eyes and squeeze their hand). Apart from propofol, no sedative drugs other than opioids were allowed during the 8 hours preceding the measurements or during the study period (Table 1).

### Electroencephalogram and event-related potential recording

The EEG signal was recorded using Ag/AgCl electrodes placed on the scalp according to the international 10–20 system. Two electrode locations (frontal [Fz] and central [Cz]) were used. Both electrodes were referred to the right mastoid, and the electrode–skin impedances were kept below 5 kO. The EEG signal was amplified and digitized continuously at 279 Hz using EMMA (ERP measuring machine; developed and custom-made in the Department of Clinical Neurophysiology, Kuopio University Hospital, Kuopio, Finland).

Background EEG was recorded for 5 min during sleep and/or while the patients lay motionless with their eyes closed. Auditory stimulation was then set to 'on' so that ERPs could be recorded. The stimulation was applied according to an oddball paradigm, which consisted of 85% standard (800 Hz) and 15% deviant (560 Hz) stimuli, with an interstimulus interval of 1 s. The duration of each stimulus was 84 ms, including 7 ms rise and fall times. Altogether 600 stimuli were delivered through earphones to the right ear for each measurement, corresponding to a recording time of about 10 min. The stimulus intensity was set at 75 dB.

### Electroencephalogram analysis

The background EEG, measured before auditory stimulation, was band pass filtered using a finite impulse response-type filter employing cutoff frequencies of 0.5 and 32 Hz (Matlab, version 6.12; The Mathworks Inc., Natick, MA, USA). Then, the filtered EEG signal (5 min long) was cut into 5 s epochs with 50% overlap. Serious artifacts were excluded by checking the maximum amplitude for each epoch; if the amplitude was greater than 100 µV then the epoch was excluded. The appropriateness of artifact rejection was manually confirmed.

For each EEG epoch, first the root mean squared (RMS) total power was calculated. Then, the epoch was subjected to power spectral density estimation, using Welsh's averaged periodogram method [9], and the spectral edge frequency 95% (SEF95) was computed from the power spectral density using a frequency range of 0.5–32 Hz. The mean of the RMS and SEF95 values of the accepted epochs were then individually computed.

### Event-related potential analysis

The EEG signal recorded during the auditory stimulation was first filtered using a finite impulse response-type filter using cutoff frequencies of 1 and 20 Hz, and then transformed to epochs from -100 ms to +900 ms relative to the onset of each stimulus. After removing artifactual epochs (rejection level ± 100 µV), the individual responses to standard and deviant stimuli were averaged. The N100 component was defined as a maximum negative deflection appearing 80–150 ms from the stimulus onset. The amplitude and the latency of the prominent N100 components in response to standard stimuli were manually scored with respect to the pre-stimulus baseline. The MMN was obtained by subtracting first the waveform elicited by the standard stimuli from the one resulting from the deviant stimuli. The MMN was then computed from the difference curve (deviant standard) as the mean amplitude between 100 and 250 ms [10].

### Statistical analysis

We carried out exploratory analyses to determine which EEG and ERP parameters changed significantly in response to interruption to sedation. For this purpose, Wilcoxon signed rank test (nonparametric paired sample test) was applied to the N100 amplitude and latency values (in response to standard stimuli), MMN, RMS power and SEF95 values measured before and after interruption to sedation. Moreover, Kruskal–Wallis test (nonparametric counterpart of one-way analysis of variance) was used to test whether the ERP and the EEG parameters differed among the sedation levels present during propofol infusion. The effect of the total duration of propofol infusion on the studied parameters was assessed using Spearman's correlation coefficient. The recording channels Fz and Cz were studied separately. Data are expressed as mean ± standard deviation, unless otherwise indicated. All statistical analyses were done using the SPSS software (SPSS for Windows, version 11.0; SPAA Inc., Chicago, IL, USA). *P *< 0.05 was considered statistically significant.

## Results

During propofol infusion the sedation level for each patient was determined on clinical grounds. It varied from deep sedation (SAS score 2) to light sedation (SAS score 4). All patients were responsive and cooperative (SAS score 4) within 30 min after discontinuation of propofol. Weaning and extubation were successful in 10 patients, whereas sedation was electively restarted in the remaining nine patients.

Of the ERP recordings, 2% and 5% were discarded as artifact during and after sedation, respectively. Accordingly, 8% and 20% of the background EEG recordings were discarded.

### Effect of interruption to propofol infusion

**Figure 2:**
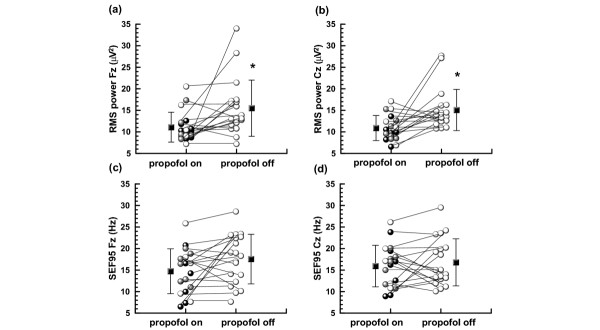
Average and individual root mean squared (RMS) power and spectral edge frequency 95% (SEF95) values during and after discontinuation of propofol infusion in the **(a, c) **frontal (Fz) and **(b, d) **central (Cz) regions. Lines connect values obtained from the same patient; black squares with vertical lines indicate the mean ± standard deviation. Individual sedation levels obtained with the Sedation–Agitation Scale (SAS): white spheres: SAS 4, gray spheres: SAS 3, black spheres: SAS2. *Significantly different from 'propofol on'.

**Figure 3:**
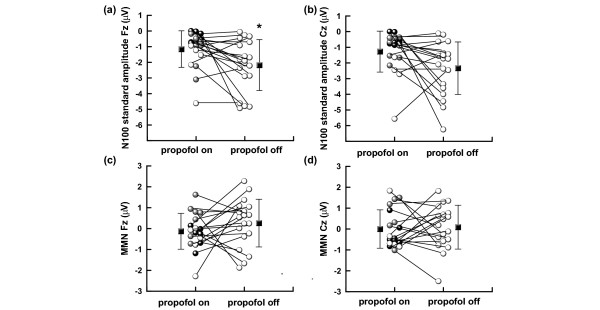
Average and individual N100 standard amplitude and mismatch negativity (MMN) values during and after discontinuation of propofol infusion in the **(a, c) **frontal (Fz) and **(b, d) **central (Cz) regions. Lines connect values obtained from the same patient; black squares with vertical lines indicate the mean ± standard deviation. Individual sedation levels obtained with the Sedation–Agitation Scale (SAS): white spheres: SAS 4, gray spheres: SAS 3, black spheres: SAS2. *Significantly different from 'propofol on'.

The EEG parameters (RMS power and SEF95) and ERP parameters (N100 and MMN) measured before and after interruption to sedation did not differ between those patients who proceeded to weaning and extubation and those in whom sedation was restarted. The RMS power increased after interruption to sedation (Fz and Cz, *P *< 0.05; Fig. 2a,2b), whereas the SEF95 values exhibited only a tendency toward a decrease (not significant; Fig. 2c,2d). The amplitude of the N100 component (in response to standard stimuli) increased at both frontal (Fz, *P *< 0.05) and central recording sites (Fig. 3a,3b). The latency of the N100 component (in response to standard stimuli) and the MMN did not change in response to interruption to propofol infusion. The MMN mean amplitude, which should be a negative value while awake, exhibited both positive and negative values after sedation had subsided (Fig. 3c,3d).

### Effect of sedation level

During propofol infusion, seven patients were deeply sedated (SAS score 2), seven patients were moderately sedated (SAS score 3) and five patients were lightly sedated (SAS score 4). The level of sedation did not influence EEG parameters. The amplitude of the N100 component (in response to standard stimuli) differed between sedation levels (Fz and Cz, *P *< 0.05), in contrast to N100 latency and MMN (Fig. 3). Both negative and positive MMN mean amplitudes were obtained independently of sedation level (Fig. 3c,3d).

Patient characteristics and duration or rate of propofol infusion did not differ among sedation level groups.

### Effect of propofol infusion duration

None of the ERP and EEG parameters correlated with the total duration of propofol infusion.

## Discussion

ERPs have exhibited graded changes with increasing doses of sedative drugs in volunteers and surgical patients [6,7], but to date no parallel studies have been conducted in severely ill patients. We assessed ERPs together with EEG parameters in a heterogeneous group of intensive care patients under sedation with propofol. The range of doses of sedative and analgesic drugs varied widely, but despite this our preliminary data suggest an association between clinical level of sedation and neurophysiological parameters. Our main findings were that the amplitude of the standard N100 component differed among the sedation levels during propofol sedation, and that the amplitude of the standard N100 in the frontal area as well as the RMS power increased in response to interruption to propofol infusion.

We selected RMS power and SEF95 to describe the changes in the EEG spectrum related to the interruption to propofol infusion. The RMS power represents the total power of the signal and the SEF95 is the frequency below which 95% of the power in the EEG spectrum resides. Sedative doses of propofol have been shown to produce an increase in total, delta and beta activity in the EEG signal, especially in the Cz and Fz regions [11-13]. In our study the total power of the EEG signal was inversely related to sedation, increasing after interruption to propofol infusion. However, the SEF95 decreased in many patients under the same circumstances. This suggests that awakening was not paralleled by a prominent increase in the high frequency range, probably due to the decrease in beta activity related to interruption to propofol infusion. Administration of opioids might also have markedly modified the EEG pattern as compared with that observed during isolated propofol infusion.

Identifiable ERPs may indicate an increased risk for auditory perception during general anaesthesia [14,15] and a positive outcome in coma patients [16,17]. During propofol sedation, the N100 component has been reported to decrease in amplitude and to delay in latency as compared with recording before the beginning of propofol infusion [5]. As sedation subsides, the opposite (amplitude increase and latency shortening) has been observed in surgical patients recovering from postoperative propofol sedation [7]. In the present study the N100 amplitude recovered similarly as the level of sedation subsided, although the amplitude values were markedly smaller than those of the surgical patients both during sedation and after sedation had subsided. Moreover, the MMN exhibited a large inter-individual variability and many patients had a positive MMN mean amplitude (Fig. 3c,3d), suggesting that MMN was not present or could not be reliably measured. In our earlier study conducted in surgical patients [7], the MMN was present at comparable sedation levels.

The small N100 amplitude and the absence of the MMN could have resulted from the use of medication other than propofol and opioids during the study period. We cannot exclude the presence of some level of sedative potentiation or side effects resulting from this medication, which might have affected the results. In all patients benzodiazepines were discontinued for a minimum of 8 hours before measurements were taken. However, some degree of residual sedative effect due to potentially impaired metabolism might have influenced our findings. Clifford and Buchman [18] reported that the combination of benzodiazepine and fentanyl affected information processing in response to novel and standard stimuli in a different manner than the combination of propofol and fentanyl in intensive care patients. Nevertheless, both of these drug combinations globally reduced the amplitudes of the responses to all stimuli as the sedative drug dose increased, in a manner similar to that in our study. We also speculate that, because of the short time allowed after propofol discontinuation, patients were still under influence of this drug during the later measurements. Thus, ERP parameters might not have had enough time to recover, even if the patients were awake and able to follow simple commands (SAS score 4). We did not study the effect of opioids on the ERPs in more detail because subanaesthetic doses of fentanyl [19] and remifentanil [20] have been shown not to attenuate the N100 component.

In the intensive care setting, EEG parameters and ERPs are influenced not only by the administration of sedative drugs but also by the underlying illness, which may cause considerable changes in functioning of the sensory pathways [21]. Diagnosis and reason for intensive care varied considerably in our population. We excluded patients with known organic brain dysfunction from the study, but it is possible that some of the patients suffered from mild subclinical neurological deficits. However, because all patients woke up and were able to follow commands, we believe that possible brain dysfunction did not have a significant effect on our results. Moreover, no differences could be found in neurophysiological parameters between extubated patients and those whose sedation was continued electively.

The statistical methods we applied deserve comment. We conducted exploratory analyses to determine which EEG and ERP parameters changed significantly because of interruption to sedation. Performing multiple comparisons, as we did, is known to increase the risk for type I error (i.e. obtaining significant differences by chance). However, because of both the exploratory nature of our analysis and the controversy concerning the Bonferroni method, we opted not to use this adjustment [22,23]. Furthermore, the heterogeneity of our patient group limits the power of statistical analysis. To overcome this limitation, we presented individual data points and used statistical analysis only to show trends in our findings.

## Conclusion

In a group of intensive care patients, with heterogeneous diagnosis and reasons for intensive care, assessment of the level of sedation using spectral EEG alone may not be sufficiently accurate. Concomitant use of ERPs, especially the N100 component, which requires widespread activity and functional integrity of the brain, may provide better distinction between sedation levels. Neurophysiological methods may thus be useful complements to clinical sedation scales in the monitoring of sedation status over time in intensive care patients under controlled sedative drug administration.

## Key messages

• 	The EGG alone may not be sufficiently accurate in the assessment of sedation levels in intensive care unit patients.

• 	Concomitant use of ERPs, especially the N100 potential, may help to differentiate between sedation levels.

• 	Neurophysiological methods may offer a complement to clinical sedation scales in neurologically intact intensive care patients.

## Abbreviations

AEP = auditory evoked potential; Cz = central region; EEG = electroencephalogram; ERP = event-related potential; Fz = frontal region; ICU = intensive care unit; MMN = mismatch negativity; RMS = root mean squared; SAS = sedation–agitation scale; SEF95 = spectral edge frequency 95%.

## Competing interests

The author(s) declare that they have no competing interests.

## Author's contributions

HYP, SN, IK, JP and ER participated in the interpretation of the results and writing of the manuscript. HYP and SN performed data collection, data entry and statistical analysis.

## Figures and Tables

**Table 1:** Demographic data, duration and rate of propofol infusion at the time of measurements

Patient number/sex	Age (years)	Length (cm)	Weight (kg)	Diagnosis	Propofol infusion^a ^(mg/kg per hour)	Duration of infusion^b ^(hours)	Opioids^c^
1/M	59	180	86	Thoracic aorta dissection	1.63	31	Oxycodon 10 mg
2/M	53	180	130	Acute myocardial infarction	0.62	11	Fentanyl 0.100 mg/hour
3/M	47	173	68	Pneumonia and sepsis (streptococcal pneumonia)	1.18	13	Oxycodon 3 mg
4/F	47	170	68	Multitrauma (renal rupture, pelvic fracture)	1.76	20	Oxycodon 10 mg
5/M	61	169	96	Ruptured abdominal aortic aneurysm	2.08	19	Fentanyl 0.100 mg/hour
6/M	76	174	73	Acute myocardial infarction and peritonitis	2.47	66	Fentanyl 0.100 mg/hour
7/F	72	160	70	Acute lung injury and status post-AVR+CABG	1.71	10	Oxycodon 5 mg
8/M	66	176	90	Acute lung injury and status post-CABG	4.44	46	Fentanyl 0.150 mg/hour
9/M	68	162	79	Wound infection post-CABG	3.04	112	Oxycodon 35 mg
10/M	64	164	65	Peritonitis and septic shock	1.85	13	Fentanyl 0.200 mg
11/F	70	162	89	Acute myocarial infarction and status post-CABG	0.67	16	Oxacodon 5 mg
12/M	83	167	65	Peritonitis	2.15	19	Fentanyl 0.075 mg
13/M	72	176	96	Sternal dehiscence post-CABG	1.46	14	Oxycodon 36 mg
14/M	76	183	77	Acute myocarial infarction and pulmonary haemorrhage	1.04	18	Oxycodon 10 mg
15/F	71	162	58	Wound infection post-CABG+AVR	1.72	19	Fentanyl 0.150 mg, oxycodon 29 mg
16/M	77	167	73	Acute respiratory distress syndrome	2.74	69	Fentanyl 0.825 mg, oxycodon 15 mg
17/F	50	170	75	Low cardiac output (status post-CABG)	1.60	86	Fentanyl 0.150 mg/hour
18/F	59	165	60	Acute lung injury and septic shock	1.67	12	Oxycodon 3 mg
19/M	71	170	80	Acute myocarial infarction and pulmonary oedema	1.50	14	Oxycodon 18 mg
Mean	65	170	79	-	1.91	31	-
SD	11	7	16	-	0.88	29	-

^a^Rate of propofol infusion at the time of measurements. ^b^Number of hours of continuous propofol infusion before measurements. ^c^Opioid medication administered during the 12 hours before (total intravenous bolus) and/or during the measurements (infusion rate). AVR, aortic valve replacement; CABG, coronary artery bypass graft.

**Table 2:** The Sedation–Agitation Scale

Score	Clinical status
7	Dangerous agitation
6	Very agitated
5	Agitated
4	Calm and cooperative
3	Sedated
2	Very sedated
1	Unarousable

Data from Riker and coworkers [8].
